# Multi-Model Allometric Analysis in the Slipper Lobster *Evibacus princeps* from the Gulf of California

**DOI:** 10.3390/ani16111637

**Published:** 2026-05-27

**Authors:** Alma Lizeth León-Valdez, Alejo Armando Valdez-Camacho, Luis Adán Félix-Salazar, Juan Francisco Arzola-González, Jorge Payan-Alejo, Yecenia Gutiérrez-Rubio, Eugenio Alberto Aragón-Noriega

**Affiliations:** 1 Facultad de Ciencias del Mar, Universidad Autónoma de Sinaloa, Paseo Claussen S/N, Mazatlán, Sinaloa 82000, Mexico; profaalmaleons@uas.edu.mx (A.L.L.-V.);; 2 Unidad Guaymas del Centro de Investigaciones Biológicas del Noroeste, S.C. Km 2.35 Camino a El Tular, Estero de Bacochibampo, Guaymas 85454, Mexico

**Keywords:** bycatch, commercial shrimp fishery, lobster fishery, multi-model approach, Akaike Information Criterion

## Abstract

The slipper lobster *Evibacus princeps* occurs as part of the bycatch in industrial shrimp fisheries of the Mexican Pacific. Although biological information on this species is limited, the Food and Agriculture Organization (FAO) currently classifies it as a subsistence resource with potential for exploitation. Accordingly, this study aimed to provide baseline information on selected biological aspects of *E. princeps* associated with industrial shrimp fishing in the Mexican Pacific. A total of 546 individuals were collected during the 2021–2022 fishing season. Female carapace length (CL) ranged from 23.96 to 139.23 mm, whereas males ranged from 16.19 to 124.75 mm CL. The size-frequency distribution of both sexes exhibited two distinct modes, suggesting that the study area may function as a permanent habitat for the species. Morphometric relationships among total length, carapace length, carapace width, and length of the third pair of pereiopods in females and the fifth pair in males were analyzed to evaluate allometric growth. Females showed positive allometric growth, growing proportionally wider than longer, from 20 to 85 mm CL. Beyond this size, they exhibited negative allometric growth. Males displayed positive allometric growth from 16 to 41 mm CL, after which negative allometric growth occurred. These findings provide baseline population information that may support future ecological, conservation, and fisheries-related studies of the species.

## Simple Summary

This study seeks to introduce a little-known and underutilized lobster species. While spiny lobsters are highly valued in seafood markets and often command prices of several hundred Mexican pesos per dish, the Mexican slipper lobster has received little commercial recognition or demand. This study provides basic biological information for evaluating the current status of the species. The broader goal is to support sustainable management and explore its potential as a resource for direct human consumption. Specimens were collected aboard commercial shrimp trawlers operating in northwestern Mexico. Size-frequency analysis revealed two distinct size groups. This pattern suggests the coexistence of different life stages and indicates that the region may support complementary phases of the species’ life cycle. Additionally, relative growth patterns differed between sexes: females exhibited negative allometric growth, whereas males showed positive allometric growth. These findings contribute to the biological knowledge required to evaluate the species’ potential for sustainable use.

## 1. Introduction

### 1.1. General Trait of Evibacus princeps

A highly coveted marine resource for over 100 years, marine lobsters sustain one of the most profitable fishing industries in the world [1]. The superfamily Palinuroidea encompasses the families Scyllaridae (slipper lobsters) and Palinuridae (spiny lobsters). This last group is a commercially highly valuable fishery resource worldwide [2]. The family Scyllaridae comprises 20 genera and at least 89 recognized extant species [2]. Members of this family occur across broad geographic ranges, inhabiting both tropical and temperate regions. They occupy diverse habitats, including sandy, muddy, and rocky substrates, and are found at depths ranging from 2 to 90 m [3], although they are most commonly encountered between 2 and 25 m.

**Figure 1:**
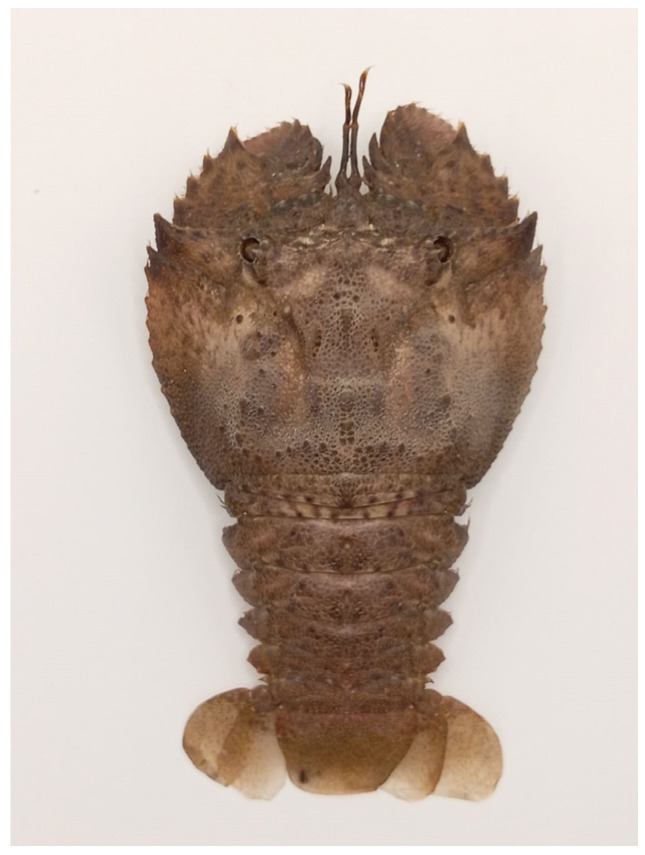
Dorsal view of *Evibacus princeps* collected from the Gulf of California.

The slipper lobster is a rare species in Mexico [4]. Within the Gulf of California, Scyllaridae is represented by only two species: *Scyllarides astori* Holthuis, 1960, and *Evibacus princeps* Smith, 1869 [4]. The latter species is distributed along coastal waters from Puerto Peñasco, Sonora, to Máncora, Peru [5]. Over time, *E. princeps* has been referred to by various common names, including shovel-nosed lobster [6], sand lobster, Chinese lobster, Pacific sand lobster [7], shield fan lobster [4], slipper lobster [8,9], and bulldozer lobster [2]. In Mexico, local fishers commonly call the species “zapatera” (shoemaker) and “cucaracha” (cockroach lobster) (Figure 1).

In Mexico, the spiny lobster fishery targeting species of the family Palinuridae represents an important component of the fisheries sector [10,11]. In contrast, members of the family Scyllaridae (slipper lobsters) constitute only a minor portion of the catch and are not considered abundant in the region. The Mexican fisheries authority (CONAPESCA) included a species-specific account for spiny lobster (*Panulirus* spp.) in the 2012 “Carta Nacional Pesquera” (National Fisheries Letter; https://www.gob.mx/cms/uploads/attachment/file/892408/CNP_2012.pdf accessed on 5 March 2026), noting that another type of lobster, slipper lobster (family Scyllaridae), occurs in the same area but that its biological characteristics are largely unknown, identifying it as a potential yet unassessed fishery resource. This situation contrasts markedly with that of Australia, where scyllarid lobsters support an established fishery with an annual catch of approximately 400 tons [12]. Nevertheless, despite the economic importance of some scyllarid species, the biology and ecology of most members of this family remain poorly documented [13]. To address this knowledge gap, a study was carried out on the somatic growth of this species along the coasts of the Gulf of California [14]. To date, this work [14] represents the first peer-reviewed publication worldwide examining any biological aspect of *Evibacus princeps*.

### 1.2. Allometric Analyses

Length–weight relationships (LWR) are widely applied in fish biology to quantify how body mass changes with length, typically expressed as a power function: *W* = *aL^b^*, where *W* is weight, *L* is length, and *a* and *b* are fitted parameters. The exponent *b* characterizes growth type: *b* = 3 indicates isometric growth, *b* > 3 reflects positive allometry, and *b* < 3 indicates negative allometry [15]. Although this model assumes that the allometric coefficient (*b*) remains constant with increasing size, even if its average may vary during ontogeny, this assumption is not always valid. In reality, *b* can change progressively or shift abruptly at specific points along the growth curve [16]. Rodríguez-Domínguez et al. [17] evaluated the traditional single power model against a multi-model framework to analyze allometry in *Callinectes bellicosus* Stimpson, 1859 from the Gulf of California. This approach has since been applied to various crustacean species, both commercial and non-commercial, such as *Pandalus platyceros* Brandt, 1851 [18] and *Calappa convexa* De Saussure, 1853 [19]. However, these studies were limited to testing models with a single breakpoint (e.g., broken-stick and two-segment models). Evidence suggests that crustacean growth may involve three distinct phases, implying the presence of two breakpoints [20]. To address this complexity, additional formulations have been developed, including three- and four-segment models, in which *b* takes different constant values (*b*_1_, *b*_2_, *b*_3_ and *b*_4_) across intervals separated by two or three breakpoints (*B*_1_, *B*_2_ and *B*_3_) [21].

Considering the above statements, the objective of the present study was to determine the most appropriate model to describe the allometric coefficient of the slipper lobster *E. princeps* in the Gulf of California using a multi-model approach.

## 2. Materials and Methods

### 2.1. Study Area and Data Collection

The Gulf of California is a semi-enclosed sea located between 22–32° N and 105–107° W. It is one of the world’s most productive marine ecosystems, with high fishing potential and exceptional biodiversity [22].

**Figure 2:**
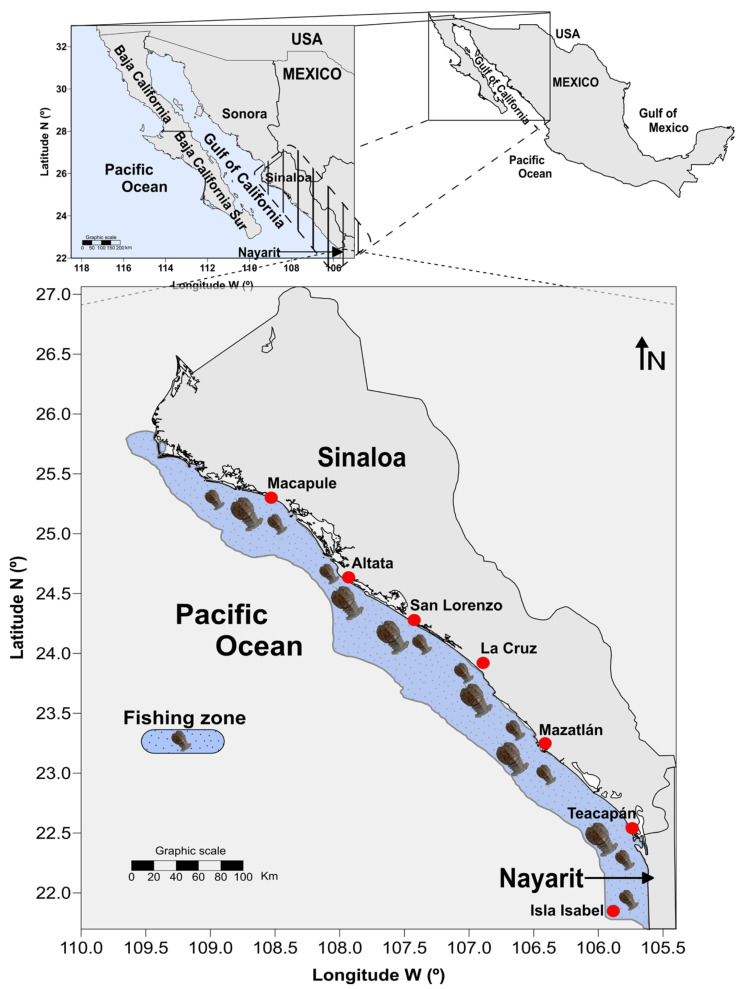
Study area in the Gulf of California.

Specimens of the slipper lobster *Evibacus princeps* were collected from the coastal region of the southern Gulf of California, spanning from Topolobampo, Sinaloa, to Isla Isabel, Nayarit (Figure 2). Sampling was carried out through bottom trawling operations conducted by the industrial shrimp fleet targeting *Penaeus* spp., based in the ports of Topolobampo and Mazatlán, Sinaloa, Mexico. All individuals were collected as bycatch (associated fauna) during the 2021–2022 shrimp fishing season (October–March) at depths fluctuating from 10 to 55 m.

Sampling was opportunistic because the species was not consistently targeted by the fishery. Consequently, extraction sites were selected by the vessel captain based on experience. Once on deck, specimens were placed in plastic bags and assigned identification numbers, which were later cross-checked with the logbook completed by the crew to record the date, trawling depth, and capture location.

Each specimen was measured, and sex was determined based on the position of the genital openings, in addition to secondary sexual traits, including the morphology of the fifth pair of pereopods and the presence of setae on the pleopods in females.

**Figure 3:**
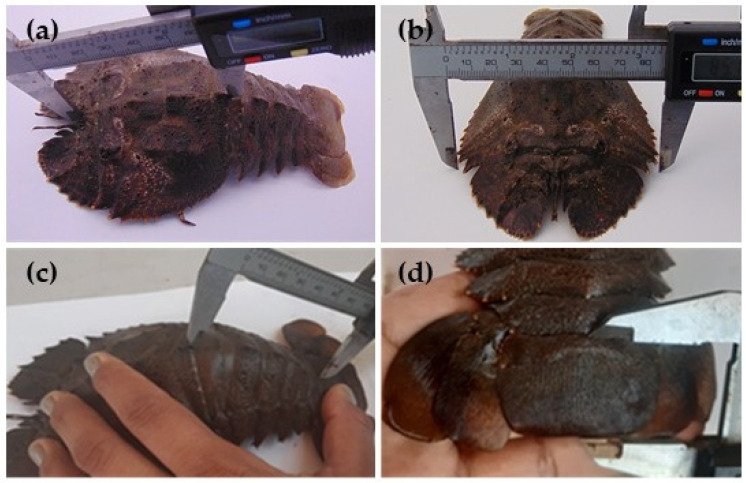
Morphometric measurements of *Evibacus princeps* caught in the Gulf of California. (**a**) carapace length (CL), (**b**) carapace width (CW), (**c**) abdominal (tail) length (AL) and (**d**) telson length.

**Figure 4:**
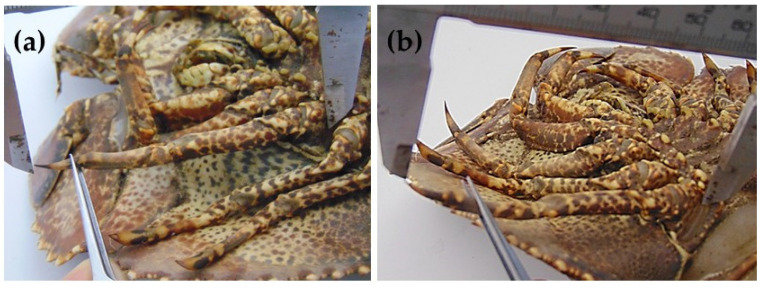
The lengths of the third (**a**) and fifth (**b**) pairs of pereiopods.

The data examined in this study come from the database previously described by León-Valdez et al. [14]. While the previous work focused on individual growth, the present research uses this dataset to perform a complementary analysis aimed at evaluating the allometric growth of *E. princeps* in relation to width carapace (WC), total length (TL), abdominal length (AL), the lengths of the third (RPL3) (Figure 3 and Figure 4) and fifth (RPL5) under a multi-model approach. The use of this database allows for maximizing the scientific value of the collected biological samples, optimizing resources, and avoiding redundant sampling efforts.

Morphometric measurements were obtained using a digital caliper with a precision of 0.01 mm. Carapace length (CL) was measured from the eye orbit to the posterior edge of the carapace of *E. princeps* (Figure 3a). Carapace width (CW) was recorded as the maximum dorsal lateral distance, measured between the third left lateral spine and the third right lateral spine of the carapace (Figure 3b). Abdominal length (AL) was measured from the first abdominal segment to the anterior edge of the telson (Figure 3c). The telson, from the anterior to posterior edge, is shown in Figure 3d. Total length (TL) was obtained by adding the measurements of CL, AL and the telson. In other words, TL = CL+ AL + telson. The lengths of the third (RPL3) (Figure 4a) and fifth (RPL5) (Figure 4b) pairs of pereiopods were measured from the proximal articulation at the base to the distal tip. Measurements were standardized by positioning the right pereiopods at a 45° angle counterclockwise and the left pereiopods at a 45° angle clockwise relative to the body axis.

### 2.2. Data Analysis

The data were first grouped by sex and then by month. Lilliefors’ test for normality and Bartlett’s test for homoscedasticity were performed using R version 4.5.1. Based on these assumptions, differences in population structure (by sex and time) were evaluated using either ANOVA or the Kruskal–Wallis test. When significant differences were detected, post hoc comparisons were conducted using Dunn’s test or Wilcoxon pairwise tests, as appropriate.

Morphometric relationships among all variables were evaluated by fitting six candidate models to log-transformed (both independent and dependent variables) data, using carapace length (CL) as the reference (independent) variable. These models were applied to examine allometric patterns between CL and the selected traits, specifically total length (TL), carapace width (CW), right propodus length of the third pereopod (RPL3), and right propodus length of the fifth pereopod (RPL5). The following equations are described for CL-TL, CL-CW, CL-RPL3 and CL-RPL5 relationships:

(1) $$\mathrm{Linear}\;(L):\;Ln\left(Y\right)=a+b_{1}Ln\left(CL\right),$$

(2) $$\mathrm{Quadratic}\;(Q):\;Ln\left(Y\right)=a+b_{1}LnCL+b_{2}Ln{\left(CL\right)}^{2},$$

(3) $$\mathrm{Cubic}\;(C):\;Ln\left(Y\right)=a_{1}+b_{1}Ln\left(CL\right)+b_{2}{Ln(CL)}^{2}+b_{3}{Ln(CL)}^{3},$$

Broken stick (BS): *Ln*(*Y*) = a_1_ + b_1_*Ln* (C*L*) if C*L* ≤ B and                 *Ln*(*Y*) = a_1_ + b_1_ *Ln* (C*L*) + (b_1_ − b_2_) *Ln*(B) if C*L* > B (4)

Two segments (TS): *Ln*(*Y)* = a_1_ + b_1_ *Ln* (C*L*) if C*L* ≤ B and         *Ln*(*Y*) = a_2_ + b_2_ *Ln* (*CL*) if C*L* > B, (5)

(6) $$\begin{array}{cc} \mathrm{Three}\;\mathrm{segments}\;(\mathrm{ThS}): & \;Ln\left(Y\right)=a_{1}+b_{1}\;Ln\left(CL\right)\;if\;CL<B_{1},\\ & Ln\left(Y\right)=a_{2}+b_{2}\;Ln\left(CL\right)\;if\;B_{1}<CL>B_{2}\mathrm{and}\\ & Ln\left(Y\right)=a_{3}+b_{3}\;Ln\left(CL\right)\;if\;CL>B_{2}, \end{array}$$

In these models, Y denotes the dependent variable (TL, CW, RPL3, or RPL5), while carapace length (CL) is treated as the independent variable. Parameters a and b correspond to the regression intercept and slope, respectively (extended as a_1_, a_2_, a_3_, b_1_, b_2_, b_3_ where applicable). The parameter B represents the breakpoint at which two linear segments with distinct slopes intersect [17], and in multi-phase models, breakpoints are defined such that B_1_ < B_2_.

The linear model assumes a constant allometric coefficient (b = b_1_) across the entire size range. In contrast, the quadratic and cubic formulations allow b to vary continuously with size (i.e., b = b_1_ + 2b_2_L and b = b_1_ + 2b_2_ logL + 3b_3_ (logL)^2^, respectively). Piecewise approaches, such as the broken-stick and two-segment models, assume two distinct constant values (b_1_ and b_2_) on either side of a single breakpoint (B). More complex representations, such as the three-segment model, incorporate two breakpoints (B_1_ and B_2_), resulting in three intervals characterized by different constant coefficients (b_1_, b_2_, and b_3_).

Quadratic and cubic models were fitted using second- and third-order polynomial regressions, respectively. All models were estimated under the assumption of additive error, and parameters were obtained through an iterative procedure based on Newton’s method to maximize the log-likelihood function [19]:

(7) $$LL\left(\emptyset |data\right)=\;\left(-\frac{n}{2}\right)\left[Ln\left(2\pi \right)+2Ln\left(\sqrt{\frac{\sum {\left(Ln(y/\;\hat{y}\right)}^{2}}{n}}\right)+1\right],$$

The breakpoint in the BS and TS models was estimated and validated using a likelihood-based search and response surface analysis. For the broken-stick (BS) and two-segment (TS) models, separate linear regressions were fitted on either side of candidate breakpoints. The breakpoint, constrained within the observed range of the independent variable, was identified by systematically scanning this range and selecting the value that maximized the log-likelihood function. This procedure was implemented using the What-If Analysis tool in spreadsheet of software Excel^TM^ Microsoft 365 (WA, USA). Because numerical optimization may place the breakpoint at points of variance change, potentially leading to false maximum likelihood estimates, a response surface analysis was conducted in Excel to validate the selected breakpoint. A similar approach was applied to the three-segment (ThS) model, in which three linear components were fitted: one before, one between, and one after two breakpoints estimated simultaneously.

Akaike’s Information Criterion (AIC) was used as a model selection criterion to select the most appropriate model [23]. The lowest value of AIC corresponds to the best model according to the following expression:

(8) $$AIC=-\;2\left(LL\right)+2\emptyset ,$$

Here, ∅ denotes the number of parameters in each model plus one additional term to account for normally distributed errors with constant variance, and LL represents the maximum log-likelihood. Differences in Akaike’s Information Criterion among models were computed as ΔAIC = AIC_i_ − AIC_min_. Following [23], models with ∆AIC < 2 were considered to have strong empirical support, whereas those with ΔAIC > 10 were regarded as unsupported and excluded from further consideration. To assess the relative plausibility of each candidate model, Akaike weights (W_i_) were calculated using the corresponding formulation:

(9) $$W_{i}=\;\frac{e^{\left(-0.5∆AIC\right)}}{\sum e^{\left(-0.5∆AIC\right)}},$$

These weights were interpreted as the strength of evidence supporting model *i* within the candidate set [23,24]. A weighted estimate of the allometric coefficient was then obtained by summing the products of each model-specific coefficient and its associated Akaike weight.

Given that allometric relationships generally do not remain consistent throughout the entire life cycle, but instead vary across developmental stages [25], the dataset was subdivided accordingly. Each subset was subsequently analyzed by fitting the six candidate models within a multi-model inference framework.

## 3. Results

### 3.1. Size Structure

A total of 546 specimens of *Evibacus princeps* were collected during the 2021–2022 industrial shrimp fishing season in the Pacific Northwest (Table 1). Sex was determined based on external morphological characteristics that distinguish females from males. However, sex could only be reliably identified in individuals exceeding 23.96 mm carapace length (CL). For this reason, 178 individuals were classified as undetermined sex (Table 1). Out of 546 specimens collected, only 368 were analyzed due to clear sex determination; the remaining specimens were excluded because sex could not be reliably identified. Of the analyzed individuals, 47% (173) were females and 53% (195) were males. Each age group identified in the samples was analyzed under the assumption of normality; however, a normal distribution was only confirmed for group 2 in both sexes (females: D = 0.8820, *n* = 69, *p* = 0.2029; males: D = 0.4270, *n* = 37, *p* = 0.5500). The two-sample Kolmogorov–Smirnov test revealed significant differences between female and male groups (D = 0.2828, *p* = 4.4635 × 10^−7^, α = 0.05).

**Figure 5:**
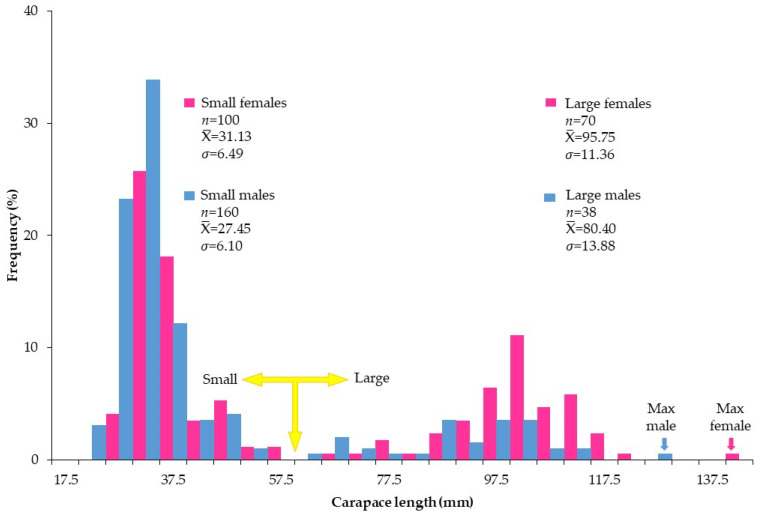
Size structure of *Evibacus princeps* Smith, 1869. Caught in the Gulf of California.

The size distribution of *E. princeps* by sex, used in subsequent analyses, is presented in Figure 5. In females, a bimodal size distribution was observed in carapace length. Sizes varied between 23.96 and 139.23 mm CL. The first mode, centered at 31.13 ± 6.49 mm CL, accounted for 60% of females, whereas the second mode, centered at 95.75 ± 11.36 mm CL, represented the remaining 40% of individuals (values are mean ± standard deviation σ). Sizes fluctuated from 16.19 to 124.75 mm CL in males. Two modes (27.45 ± 6.10 and 86.40 ± 13.88 mm) were calculated. The second mode accounted for 20% of individuals. The *t*-test revealed significant differences between the two modes within each sex (*p* < 0.00004). It is worth noting that this figure represents a modified version of the one previously published in [14]. Its inclusion here is necessary to clarify the rationale for dividing the analyses into small- and large-size groups for both sexes. This constitutes the only overlap between the two studies. Additionally, the figure presentation has been redesigned to avoid direct repetition of the previously published version [14].

### 3.2. Allometric Pattern

The coefficient of determination (r^2^) showed that, for all analyzed correlations, at least 98% of the variability in the dependent variable is explained by the independent variable. This shows a robust relationship between the variables and suggests that the derived equations provide reliable estimates for predicting one variable based on the other.

It is important to note that the data for both females and males were divided into two size groups: small and large. Evaluation of model support showed that the AICc favored the linear model, identifying it as the best fit for the CL–TL relationship in females of both groups (Table 2) and in males of the large group. In contrast, for the same relationship in the small group of males, the two-segment model provided the best fit (Table 3). For the CL–CW relationship, the linear model was the best fit for males in both size groups (Table 3) and for females in the small group (Table 2), whereas the two-segment model was favored for females in the large group (Table 2).

Additionally, evaluation of the AIC values indicates that the linear, quadratic, and two-segment models represent the best-supported models, or those with substantial support [23]. The level of evidence supporting these models ranged from 49% to 72%, as indicated by their respective Akaike weights (Table 2 and Table 3).

**Figure 6:**
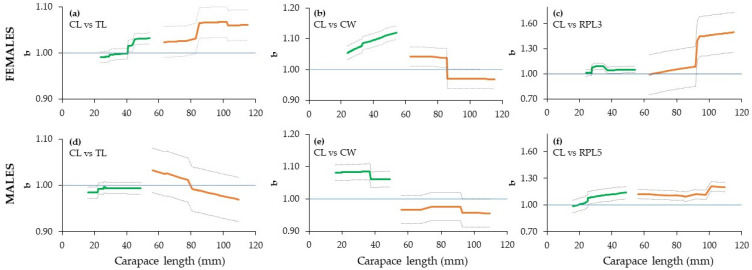
Relationship between carapace length (CL) and different morphometric variables of *Evibacus princeps.* Females (panels **a**–**c**) and males (panels **d**–**f**). The values of the allometric coefficient *b* are shown as a function of size (CL, mm) for the relationships: CL vs. TL (**a**,**d**), CL vs. CW (**b**,**e**), and CL vs. RPL3/RPL5 (**c**,**f**). The colored lines represent the estimated *b* values by size interval, while the gray lines indicate the confidence intervals (95%). The horizontal blue line at *b* = 1 denotes isometric growth; values above or below this line indicate positive or negative allometry, respectively. For each graph, the green solid line represents small organisms, and the solid orange line represents large organisms.

Females showed positive allometric growth, growing proportionally wider than longer, from 20 to 85 mm CL, followed by negative allometric growth beyond this size. Males displayed positive allometric growth from 16 to 41 mm CL, after which negative allometric growth occurred. Because no single model was clearly superior, model averaging was applied, and the resulting estimates were plotted. Figure 6 illustrates differences in growth patterns between females and males, as well as distinctions between the small and large size groups within each sex.

**Figure 7:**
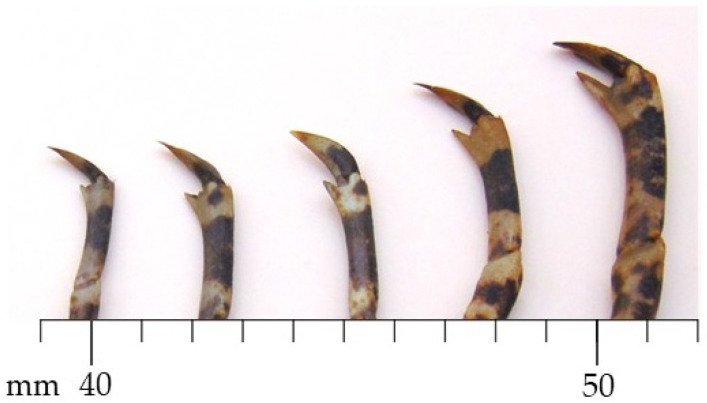
Development of the distal claw of the fifth pair of pereiopods in female *E. princeps* from 39 mm to 52 mm CL.

In females, all graphs show a breakpoint between 35 and 40 mm in CL, which coincides with the development of the distal claw (Figure 7). In the females examined in this study, this structure began to develop at about 39 mm CL.

**Figure 8:**
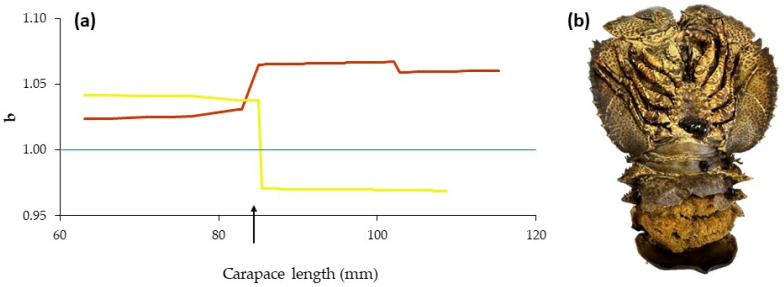
Relative growth of *Evibacus princeps* Smith, 1869. (**a**) Relationship between carapace length and total length CL–TL (red line) and carapace width CL–CW (yellow line) highlighting the breakpoint, which closely corresponds to the smallest ovigerous female recorded (86 mm CL). The horizontal blue line at *b* = 1 denotes isometric growth; values above or below this line indicate positive or negative allometry, respectively. (**b**) The smallest ovigerous female recorded in this study.

In Figure 8, the CL-TL and CL-CW relationships were extracted from Figure 6 to highlight that the breakpoint matches the smallest ovigerous female collected during the survey. The biological explanation of the breakpoint is difficult to establish, but it is possible to relate the breakpoint of two biometric relationships such as CL-TL and CL-CW to the ovigerous females, as the smallest female found in the present study clearly matches at 86 mm of CL.

## 4. Discussion

The primary purpose of fitting biological observations to mathematical models is to determine which model most accurately represents the underlying biological pattern. Practically, this process allows researchers to evaluate whether the data conform more closely to one biological interpretation rather than another. Studies on several marine organisms [16] demonstrated that ontogenetic growth patterns can vary considerably: in some body parts, growth follows a constant allometric exponent, whereas in others the allometric coefficient changes gradually, or exhibits abrupt shifts marked by breakpoints. These findings suggest that models other than the traditional power function may be more appropriate for describing allometric growth [16].

For fish length–weight relationships analyses, separating datasets by sex and maturity stage and fitting a power model to each subset was recommended [26]. In contrast, the present study implemented a multi-model inference framework and used one of the two equations that were introduced as additional equations to describe allometric growth [21]. A key advantage of this approach over reliance on a single model is that it enables hierarchical ranking of candidate models according to their relative support. When no single model clearly dominates, defined as a model weight greater than 90% [23], information from multiple models can be integrated to produce an averaged representation of the biological process being analyzed. In the present study, the selection of the most appropriate model is summarized in Table 2 and Table 3 for females and males, respectively (also including small and large sizes). Unlike the previous growth analysis by León-Valdez et al. [14], which was based exclusively on carapace length, in this study a total of 12 allometric relationships were evaluated. The linear model was identified as the best-fitting model in eight cases and ranked as the second-best in one case. However, the highest level of support for this model reached only 67% in large females. This indicates that no single model clearly dominated; therefore, a model-averaging approach was applied to obtain a representative description of the relationships analyzed.

The two-segment model was identified eight times as either the best or the second-best-fitting model. This model incorporates a single breakpoint, assuming that the allometric coefficient (b) has b_1_ and b_2_ as constant values at both sides of the breakpoint (B). The broken-line and two-segment models, therefore, describe growth as two linear phases with different slopes separated by this transition point.

The quadratic model ranked third in overall performance, as it was selected as the best or second-best model in six cases. Unlike the segmented models, the quadratic formulation assumes that the allometric coefficient (b) varies continuously with size.

The averaged model is illustrated in Figure 6. In small females, particularly in the CL–CW relationship, and in large males in the CL–TL relationship, the gradual variation in b values (allometric coefficient) with size is clearly evident. Nevertheless, the most pronounced feature in Figure 6 is the presence of a breakpoint separating two linear trends with distinct slopes. This abrupt shift in the relationship between structures, specifically relative to carapace length (CL), appears to be the most characteristic growth pattern observed in the slipper lobster population from the Gulf of California.

The three-segment model proposed as a novel approach for analyzing allometric growth in blue shrimp [21] was only selected as the second-best model for the CL–RPL3 relationship in small females in the present study, with 27% of the supporting evidence. This contrasts with the results reported by Leyva-Vázquez et al. [21], who frequently identified the three-segment model as the best model in their analyses, with more than 90% of the supporting evidence. In the case of the slipper lobster, however, this model did not adequately describe the observed allometric relationships. This observation is not intended as a criticism of their study but rather indicates that the proposed equation may not perform equally well across different decapod species. Notably, Leyva-Vázquez et al. [21] also reported that the linear model was never adequate, ranking last among the 12 datasets analyzed in their study. This is contrary to the results of the present study, in which the linear model was best or second-best in nine cases.

The observed size distribution likely reflects the extensive spatial coverage of the study area, which prevented detection of any clear relationship between latitude and body size. The presence of two well-defined modes in both sexes indicates that the region may function as a resident habitat for *E. princeps*. These findings highlight the need for targeted research efforts focused on this species, including the incorporation of trained personnel aboard shrimp trawlers to improve data collection.

The coefficient of determination (r^2^) indicates that, in all correlations analyzed, at least 0.98 of the variation in the dependent variable is explained by the independent variable. This suggests that the resulting equations provide reliable estimates for predicting one variable from the other. The coefficient of determination has also been widely used to describe linear relationships in numerous crustacean species, including the box crab *Calapa convexa* [27], lobsters of the genus *Panulirus* [28,29], and species of the family Scyllarides [30,31,32].

The growth pattern of *E. princeps* was found to vary according to developmental stage, differing from that of *Scyllarides deceptor*, which shows no morphometric differences between juveniles and adults [32]. In some males, a sperm mass was observed emerging from the genital opening beginning at approximately 91 mm carapace length (CL). This observation coincides with the breakpoint shown in Figure 6, a pattern similar to that reported for male *S. latus* in the Mediterranean [33]. The same breakpoint at 91 mm CL was also identified in the relationships between CL and LA, LPD3, and LP5 on both sides, where the male genital openings are located.

The sudden shift in relative growth observed during the transition from juvenile to adult stages in crustaceans has been attributed to the reallocation of energy toward gonadal development. Consequently, adults tend to exhibit lower relative weight growth compared with juveniles, as a greater proportion of energy is directed to reproductive maturation rather than somatic growth. In females, as illustrated in Figure 8, the detected breakpoint corresponds to the size at which the smallest ovigerous female recorded in this study was observed. The biological significance of this breakpoint remains uncertain, and therefore, this result should be interpreted with caution. Further research based on larger sample sizes will be necessary to clarify this pattern. However, it is important to note that previous investigations have reported limited specimen availability, which has constrained more robust analyses. For instance, Tirado-Ibarra et al. [34] reported capturing only a single *E. princeps* specimen during 12 commercial shrimp fishing trips.

As a secondary morphological characteristic, the presence of a distal claw on the fifth pair of pereiopods was observed in females (Figure 7) that had not yet reached full development. This feature was first detected in females from 39 mm CL onward and frequently appeared, or reached full development, earlier than the setae on the pleopods. The distal claw is presumed to function in breaking up the spermatophore mass. The progression of this morphological characteristic is documented here, supported by the large number of specimens collected during the present study.

It is important to acknowledge several limitations of this study. First, samples were obtained from a commercial fishery in which the slipper lobster is not a target species but occurs as bycatch. Second, specimens were collected across multiple locations, and the sampling period extended over a relatively long timeframe, both of which may introduce additional variability. This spatiotemporal dispersion introduced variability that could not be controlled with the current design. Therefore, regarding the methodological approach, it is recommended to complement this work with a comprehensive analysis of mitochondrial markers to more accurately assess genetic variability, population structure, and optimal conditions for growth and survival, such as that carried out by Soberanes-Yepiz et al. [35] for *Macrobrachium americanum*; Mancuso et al. [36] for *Callinectes sapidus*; and Spencer et al. [37] for spiny lobster *Panulirus ornatus.*

Despite these constraints, the dataset remains valuable, as it represents the most comprehensive information compiled for this species to date. Previous studies of bycatch in shrimp fisheries have reported extremely limited records, in some cases documenting only a single specimen per fishing season [34], while onboard observer programs have recorded as few as two individuals over a 10-year period. In this context, the present study provides a meaningful contribution and an important baseline for future research.

## 5. Conclusions

The occurrence of *Evibacus princeps* in the industrial *Penaeus* spp. fishery of the Mexican Pacific confirms that this slipper lobster inhabits the continental shelf of the Gulf of California. It occurs across multiple developmental stages (larvae, juveniles, and adults) at depths of 10–50 m. Its distribution from the Port of Topolobampo to Isla Isabel National Park indicates a broad regional presence. This suggests that the species forms part of the benthic community affected by the shrimp trawl fishery.

Morphometric analyses suggest that *E. princeps* generally follows an isometric growth pattern; however, multi-model evaluation of the carapace length–carapace width relationship revealed positive allometric growth in both sexes, indicating proportionally greater increases in width than length during development. These results demonstrate that multi-model approaches provide a more robust framework for detecting subtle growth patterns than single-model analyses, thereby improving the reliability of morphometric assessments in crustacean populations. We concluded that multiple models were necessary to determine whether growth showed positive or negative allometry, as it initially appeared to be isometric.

As the first population-level study of *E. princeps* in this sector of the Mexican Pacific, this research provides essential baseline information on its distribution and growth dynamics. Such knowledge is critical for evaluating the ecological role and fishery interactions of this species and may serve as a scientific basis for future monitoring programs, bycatch assessments, and management strategies aimed at promoting the sustainable use of marine resources in the region.

## Figures and Tables

**Table 1:** Industrial shrimp fishing sites for *Penaeus* spp. in the Gulf of California during the 2021–2022 season, where *Evibacus princeps* was recorded as bycatch. In the table, females captured per month at each site are denoted by “F”, males by “M” and undetermined sex by “I”.

	Fishing Sites																				
	Macapule			Altata			San Lorenzo			La Cruz			Mazatlán			Teacapán			Isla Isabel		
Sampling Months	F	M	I	F	M	I	F	M	I	F	M	I	F	M	I	F	M	I	F	M	I
October 2021	4	1	0	2	1	0	7	2	0	1	3	0	4	2	0	13	5	0	0	0	0
November 2021	2	1	1	2	2	1	2	1	0	2	0	1	5	2	1	8	15	8	4	0	1
December 2021	4	2	1	2	1	0	2	0	0	2	1	1	1	3	1	13	10	48	5	9	23
January 2022	1	1	0	33	30	16	7	12	7	1	5	3	1	1	0	1	1	1	1	0	0
February 2022	3	13	8	8	12	6	6	7	9	10	40	30	1	1	0	1	0	2	1	0	0
March 2022	10	7	7	1	2	2	0	0	0	0	0	0	1	1	0	1	1	0	0	0	0

**Table 2:** Models adjusted to the CL-TL, CL-CW and CL-RPL3 data of *Evibacus princeps* in small and large sizes for females. For each relationship, the lowest AICc indicates the best model (blue-colored numbers). The numbers in green represent those models with substantial support [23]. AICc is the Akaike value. ΔAICc is the difference in Akaike values. W_i_ is the Akaike weight.

Morphometric Relationship	Model	AICc	ΔAICc	W_i_	AICc	ΔAICc	W_i_
		Small Size Group			Large Size Group		
CL-TL	Linear	−606.99	0.00	0.39	−360.25	0.00	0.39
	Quadratic	−604.60	2.39	0.12	−358.18	2.07	0.14
	Cubic	−602.79	4.19	0.05	−355.85	4.40	0.04
	Broken stick	−603.52	3.46	0.07	−356.27	3.98	0.05
	Two segments	−606.49	0.49	0.30	−359.92	0.33	0.33
	Three segments	−603.77	3.21	0.08	−355.29	4.96	0.03
CL-CW	Linear	−462.15	0.17	0.30	−328.01	2.46	0.20
	Quadratic	−462.32	0.00	0.32	−325.94	4.53	0.07
	Cubic	−460.07	2.25	0.11	−324.06	6.41	0.03
	Broken stick	−460.29	2.03	0.12	−324.84	5.63	0.04
	Two segments	−460.07	2.25	0.10	−330.47	0.00	0.67
	Three segments	−458.62	3.70	0.05	−355.29	13.88	0.00
CL-RPL3	Linear	−326.75	0.00	0.30	−61.51	0.61	0.30
	Quadratic	−325.28	1.47	0.14	−60.09	2.04	0.15
	Cubic	−322.99	3.76	0.05	−57.46	4.67	0.04
	Broken stick	−323.70	3.05	0.06	−58.58	3.54	0.07
	Two segments	−325.75	0.99	0.18	−62.12	0.00	0.41
	Three segments	−326.55	0.20	0.27	−56.58	5.55	0.03

CL carapace length, TL total length, CW carapace width, RPL3 lengths of the third pairs of pereiopods.

**Table 3:** Models adjusted to the CL-TL, CL-CW and CL-RPL5 data of *Evibacus princeps* in small and large sizes for males. For each relationship, the lowest AICc indicates the best model (numbers in blue). The numbers in green represent those models with substantial support [23]. AICc is the Akaike value. ΔAICc is the difference in Akaike values. W_i_ is the Akaike weight.

Morphometric Relationship	Model	AICc	ΔAICc	W_i_	AICc	ΔAICc	W_i_
		Small Size Group			Large Size Group		
CL-TL	Linear	−975.47	18.11	0.00	−165.10	0.00	0.46
	Quadratic	−982.93	10.65	0.00	−163.22	1.88	0.18
	Cubic	−983.17	10.41	0.00	−162.73	2.37	0.14
	Broken stick	−987.46	6.12	0.03	−162.40	2.70	0.12
	Two segments	−993.58	0.00	0.72	−161.77	3.33	0.09
	Three segments	−991.38	2.20	0.24	−156.83	8.27	0.01
CL-CW	Linear	−606.35	0.00	0.49	−145.94	0.00	0.63
	Quadratic	−604.28	2.07	0.18	−143.29	2.65	0.17
	Cubic	−602.14	4.21	0.06	−140.46	5.48	0.04
	Broken stick	−602.59	3.75	0.08	−140.74	5.20	0.05
	Two segments	−604.14	2.20	0.16	−141.23	4.70	0.06
	Three segments	−600.94	5.40	0.03	−141.01	4.93	0.05
CL-RPL5	Linear	−266.67	0.00	0.31	−89.82	0.00	0.38
	Quadratic	−265.95	0.71	0.22	−88.43	1.39	0.19
	Cubic	−263.81	2.85	0.08	−84.43	5.39	0.03
	Broken stick	−265.25	1.41	0.15	−85.81	4.01	0.05
	Two segments	−265.84	0.83	0.21	−89.42	0.40	0.31
	Three segments	−262.12	4.54	0.03	−85.54	4.28	0.04

CL carapace length, TL total length, CW carapace width, RPL5 lengths of the fifth pairs of pereiopods.

## Data Availability

All data analyzed and generated by this study are available upon reasonable request to the corresponding author.

## Abbreviations

The following abbreviations are used in this manuscript:

AIC	Akaike Information Criterion
AL	Abdominal (tail) length
CL	Carapace length
CW	Carapace width
FAO	Food and Agriculture Organization
CONAPESCA	National Fisheries and Aquaculture Commission
RLP3	The lengths of the third pair of pereiopods
RLP5	The lengths of the fifth pair of pereiopods
TL	Total length
